# Home Caregivers of Elderly People: Perceptions and Quality of Life

**DOI:** 10.3390/geriatrics10030061

**Published:** 2025-04-29

**Authors:** Luís Eduardo Genaro, José Victor Marconato, Aylton Valsecki Júnior, Tânia Adas Saliba, Fernanda Lopez Rosell

**Affiliations:** 1 Postgraduate Program in Collective Health in Dentistry, School of Dentistry, São Paulo State University, Araçatuba 16.015-050, São Paulo, Brazil; 2 School of Medicine, San Francisco University, Bragança Paulista 12.916-900, São Paulo, Brazil; jose.marconato@mail.usf.edu.br; 3 Department of Community Dentistry, School of Dentistry, São Paulo State University, Araraquara 14.801-903, São Paulo, Brazil; 4 Department of Preventive and Restorative Dentistry, School of Dentistry, São Paulo State University, Araçatuba 16.015-050, São Paulo, Brazil

**Keywords:** home care, elderly, caregivers

## Abstract

**Objective:** In this study, we aimed to identify the main factors that influence the quality of life of caregivers in the context of home care for the elderly. **Methodology:** This is a mixed-methods study with a qualitative–quantitative approach, conducted with 138 home caregivers from the city of Itatiba, São Paulo, Brazil. Individual interviews were conducted, and the qualitative data were analyzed using the Collective Subject Discourse technique. Simultaneously, the quantitative approach involved the application of the EQ-5D questionnaire to assess health-related quality of life, and the data were analyzed using descriptive statistics and significance tests. **Results:** The majority of caregivers were female, accounting for 92.03% of the total, with the predominant age group being over 50 years old (49.28%). The interviews highlighted the regularity of home visits by healthcare professionals, emphasizing the importance of these visits for the continuity of treatment at home. However, some caregivers expressed feelings of loneliness due to social isolation and emotional burden, reporting difficulties in resting at night and experiencing pain. In the quality of life assessment, statistically significant differences were identified in various dimensions of the EQ-5D. Women showed a higher proportion of extreme problems in usual activities (*p* < 0.001) and pain/discomfort (*p* = 0.02), while men reported more moderate problems with anxiety/depression (*p* = 0.03). **Conclusions:** This study highlights the importance of personalized and accessible care for patients. It underscores the need for emotional support and educational resources for caregivers to mitigate the negative impacts of prolonged caregiving on their physical and emotional health.

## 1. Introduction

Globally, the number of individuals aged 65 and older is expected to double between 2000 and 2030, with an average life expectancy approaching 80 years [1,2]. Consequently, as life expectancy increases, the number of older adults requiring long-term home care also rises [1,3].

The caregiving role constitutes a risk factor for caregiver mortality, and those experiencing excessive burden exhibit significantly higher levels of depressive symptoms, anxiety, and a less favorable perception of their own health [4]. Caregiver health is of utmost importance for patients, as the risk of the institutionalization of older adults is closely linked to the deterioration of their caregiver’s health [2]. Protective factors, such as social and emotional support, play a crucial role in assisting caregivers by reducing stress and burden [2,5,6,7].

Caregivers are defined as either paid professionals or family members who provide assistance to individuals with chronic or disabling conditions [7]. They play a vital role in caring for individuals who are ill or incapable of fulfilling their own needs [7,8]. Given this dynamic, the physical and emotional health of caregivers has emerged as a growing public health concern [2,9].

Caregiving responsibilities significantly impact the quality of life of family caregivers. Many caregivers, due to the demands of their role, experience sleep disturbances that negatively affect both sleep quality and overall physical and mental health [10]. Caregiving tasks are often time-consuming, limiting caregivers’ opportunities to engage in leisure activities, with more than half of family caregivers sacrificing social interactions due to their caregiving responsibilities [10,11].

This situation has a substantial influence on the social lives of family caregivers, restricting their participation in social events and interactions with family and friends [12]. Therefore, it is essential to provide effective strategies for both family caregivers and care recipients to enhance the latter’s independence while reducing both caregiver burden and time spent on caregiving tasks. Implementing improvements and interventions to create an accessible home environment represents a key strategy to address this issue [13].

In Brazil, home care has become a key strategy within the Unified Health System. A 2020 exploratory study analyzed the implementation and use of these services, revealing that between 2008 and 2016, 94,754 home hospitalizations were recorded, and from 2012 to 2016, a total of 4,008,692 home-based outpatient procedures were performed [14]. This study also highlighted significant regional disparities, indicating inequalities in the access and availability of home care services. Furthermore, a 2016 survey on the prevalence of home care among the elderly population in Brazil found that 11.7% of older adults received such care. Factors such as advanced age, lower educational attainment, and lower socioeconomic status were associated with a higher likelihood of receiving home care [15].

Research involving home caregivers is crucial given the global demographic aging trend and the increasing demand for long-term home care. As life expectancy rises and the number of older adults requiring assistance grows, understanding the physical and emotional impacts on caregivers becomes imperative. Thus, the objective of this study was to identify the main factors that influence the quality of life of caregivers in the context of home care for the elderly.

## 2. Methodology

### 2.1. Study Design and Sample Selection

This is a mixed-methods, cross-sectional study combining both quantitative and qualitative components to better understand caregivers’ quality of life and their perceptions regarding home care provided in primary healthcare.

The participants in this study, which focused on caregivers’ perceptions regarding home care in primary healthcare, were selected through purposive sampling. Initially, all home caregivers in the specific area were invited to participate, totaling 290 caregivers. Contact was established via telephone. Of the 290 invitees, 138 agreed to participate. This recruitment process involved identifying potential participants based on predefined inclusion criteria: being 18 years or older and responsible for providing home care to a dependent individual within the coverage area of the local Family Health Strategy (FHS) team. Those who demonstrated interest in participating contacted the research team directly. All eligible participants received detailed information about the study, as well as a consent form for participation.

### 2.2. Study Location

This research was conducted in the city of Itatiba, located in the state of São Paulo, Brazil, classified as a medium-sized municipality. Itatiba is part of the Metropolitan Region of Campinas and is situated northwest of the state capital, approximately 80 km away.

### 2.3. Research Ethics

The research procedures were reviewed by the Research Ethics Committee on Human Subjects at the School of Dentistry of Araraquara, São Paulo State University (UNESP) (CAAE: 69122923.6.0000.5416, approved on 13 June 2023). This study followed the ethical protocols and adhered to the guidelines for both qualitative and quantitative components. This qualitative study followed the COREQ criteria (Consolidated Criteria for Reporting Qualitative Research), while the quantitative portion followed the STROBE guidelines. Participation in this study was voluntary, and written informed consent was obtained from all participants.

### 2.4. Data Collection

A trained researcher conducted the interviews using a semi-structured script composed of a series of open-ended questions and quality of life questionnaires, aiming to comprehensively explore participants’ perspectives and experiences. Each interview was conducted individually in a quiet and comfortable environment to ensure participant privacy, lasting approximately 60 min. All interviews were recorded to ensure accuracy in terms of transcription and data analysis. During the interviews, the researcher maintained a neutral stance, allowing the participants to freely express their opinions and experiences. After the interviews were completed, the recordings were transferred from the recorder to a computer for audio transcription and subsequent analysis.

### 2.5. Discourse of the Collective Subject (DCS)

The analysis of qualitative data was conducted using descriptive techniques and the Discourse of the Collective Subject (DCS) methodology. As described by Lefevre et al. [16], this approach is grounded in the Theory of Social Representations, incorporating sociological principles and examining the verbal narratives collected during this study. This method allows for individual statements to be synthesized into a representative expression of collective thoughts based on the understanding that, within a social group, individuals share beliefs, ideas, and opinions on specific topics.

In this process, the various individual narratives are combined into a single collective discourse, which reflects the commonalities among participants. The main source for constructing this collective discourse is the content of the interviews. To create a sense of unity in the collective thinking, the discourse is written in the first-person singular, as if spoken by a single social subject, even though it represents a collective. This construction is made possible by the theoretical foundation of Social Representations, which views discourse as an externalization of social experiences internalized by individuals [17,18].

Following data collection, each individual statement was carefully analyzed to build the collective thought. The initial step involved identifying key expressions, continuous or discontinuous excerpts from the individual narratives that reveal the essence of the content. These expressions were rigorously selected to ensure that only the most representative elements were preserved, avoiding both excessive inclusion and the omission of relevant information.

Next, central ideas were derived from the key expressions, representing the core meaning of what the interviewees intended to convey. When central ideas were found to be similar or complementary, they were grouped into common categories, each corresponding to a specific question in the interview guide.

Finally, based on the key expressions and the central ideas of each category, a synthesized discourse was written in the first-person singular, representing the DCS. This discourse summarizes the participants’ collective perception, as if all were expressing the same opinion through a single voice. One DCS was constructed for each category identified throughout the study [18].

### 2.6. Health-Related Quality of Life Measured with EQ-5D

The EQ-5D assessment involves analyzing five main dimensions of an individual’s health status: mobility, self-care, usual activities, pain/discomfort, and anxiety/depression. Each of these dimensions presents three response levels, indicating the presence of problems at different degrees [19]. Participants are asked to provide responses for each dimension, assigning a numerical code to their condition. For example, code 1 may represent “no problems”, code 2 may indicate “some problems”, and code 3 may correspond to “severe problems”. The combination of these codes across the five dimensions generates a unique health profile for each individual [20].

The version used was the EQ-5D-3L translated and validated for the Brazilian population. Its cross-cultural adaptation followed standardized procedures, and reliability was previously confirmed in studies with Brazilian caregivers. Internal consistency and construct validity have been supported in the literature for similar populations [21].

### 2.7. Health State Valuation

The EQ-5D employs two approaches to assess the valuation of an individual’s health state. First, to complement the description of health status, it offers respondents the opportunity to position their own health state on a visual analog scale. Through the direct measurement technique, participants are invited to draw a line representing their current health state and the EQ-VAS thermometer, which ranges from 0 to 100. On this scale, 0 represents the worst imaginable health state, and 100 represents the best imaginable health state [20].

### 2.8. Data Analysis

Qualitative and quantitative data were analyzed separately and then integrated at the interpretation stage. This is characteristic of the convergent parallel mixed-methods design.

For the qualitative data, interviews were first fully transcribed. Then, key expressions and central ideas were extracted to construct synthetic discourses using the Discourse of the Collective Subject method. Two independent researchers coded and categorized the data, with a third reviewer resolving discrepancies. Rigor was ensured through methodological triangulation, peer debriefing, and participant feedback on preliminary interpretations.

Quantitative data underwent a normality test using the Shapiro–Wilk test. Due to the lack of normal distribution, the Chi-square test was performed for evaluation. A significance level of 0.05 was adopted for all of the analyses conducted. Statistical analyses were performed using IBM SPSS Statistics 19.0 (IBM Corp., Armonk, NY, USA). To ensure ethical integrity, participants were identified in the results as “P” followed by a cardinal number.

The integration of the data occurred after separate analyses were completed. The findings from the EQ-5D were compared and contrasted with the themes that emerged from the qualitative interviews, allowing for a comprehensive understanding of caregiver burden and satisfaction with home care services.

## 3. Results

### 3.1. Sociodemographic Profile and Caregiving Experience

Table 1 provides an overview of the sample distribution across different demographic and health variables.

Regarding gender, the majority of caregivers are female, accounting for a significant 92.03% of the total, while males represent only 7.97%. In terms of age group, most caregivers are over 50 years old, comprising a substantial 49.28% of the total. The age groups of 31 to 35 years and 40 to 50 years are also relevant, representing 15.94% and 19.57%, respectively. Regarding race/ethnicity, the majority of caregivers are White, making up 56.52% of the total.

Concerning the relationship with the patient, most caregivers have a specific connection, with children forming the largest group, representing 44.93% of the total. Regarding the length of the caregiving experience, a diversity of experiences is observed, with approximately 34.78% having more than four years of experience.

### 3.2. Emerging Themes from Interviews on Home Care

The interviews, in turn, revealed a range of themes related to home care. Table 2 presents these themes along with their respective definitions.

Most caregivers described a consistent pattern in which home visits are conducted weekly by community health professionals, nurses, and physicians. Many shared accounts highlighting this regularity, such as the following:

*“It is a routine to see the health center professionals here every week; they rarely miss a visit” (P. 121), or also, “The health center professionals always show up every Thursday” (P. 08).*

Furthermore, other relevant aspects of home care emerged, particularly highlighting the positive factors that contribute to the continuity of treatment at home. Many study participants emphasized the importance of these visits, citing examples such as the following:

*“It is a relief to have them around; they always offer help when we need it. I remember a time when my father fell and injured his leg; the nurse came here to dress his wounds” (P. 37). Another patient added: “Their presence at home is very important because my mother cannot walk to the health center since she is bedridden” (P. 42).*

Some caregivers also highlighted the attention and care provided by the professionals during home visits, emphasizing the human connection and the sensitivity demonstrated in their roles, with comments such as the following:

*“They are very polite, always treating us with kindness” (P. 09), or “Sometimes, they seem like part of the family; I can even send a message on WhatsApp, and the nurse is always there to help” (P. 11).*

When asked about their work, some participants responded that “loneliness” is the main cause of dissatisfaction. This feeling was defined by the distancing from people and the lack of social life, leading to emotional overload, feelings that harm their quality of life.

*“It’s been a while since I’ve known what it’s like to go out and have fun, I have to stay the whole time taking care of him” (P. 15), “My friends invite me to go out, but with my father bedridden like this, it’s impossible” (P. 13), another participant even stated: “Look, I want to go out, travel, you know? But with my mother like this, it’s just not possible… I’m the only one left to take care of her” (P. 126).*

During the interview, we also discussed physical and emotional overload. Most caregivers reported difficulty resting due to nighttime interruptions and mentioned pain, especially in their arms, due to the transport of frail patients.

*“I can’t sleep more than three hours in a row, I always have to get my mother up to go to the bathroom, and when it’s not that, it’s the medication that I can’t neglect” (P.132), “It’s been a long time since I’ve known what rest is, I even have dark circles under my eyes, but what can I do, right?” (P. 87). Regarding the pain, the patients said: “My arm hurts a lot, but it’s because of his weight, right? I need to bathe him, change him … and he can’t support himself on anything” (P. 75), “…I live on painkillers because of the pain here” (patient pointing to their forearm) (P. 23).*

### 3.3. Analysis of Health-Related Quality of Life

Table 3 presents an analysis of the EQ-5D dimension, exploring the categories of sex, mobility, self-care, usual activities, pain/discomfort, and anxiety/depression.

**Figure 1:**
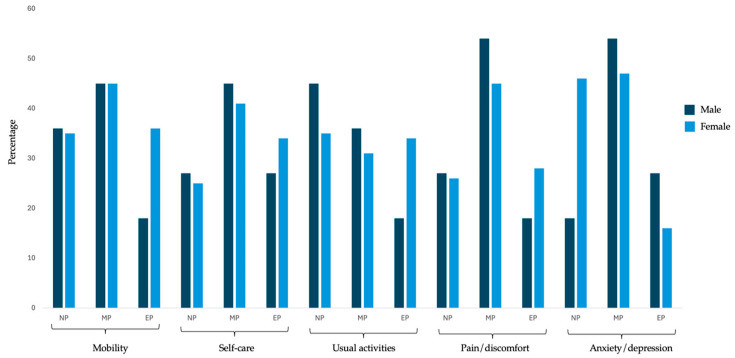
Distribution of EQ-5D dimensions according to participants’ gender.

Significant differences between the sexes were identified across several EQ-5D dimensions (Table 3, Figure 1). In terms of mobility, specifically in the category of extreme problems, a statistically significant difference was observed (*p* = 0.03). Both women and men showed a higher frequency in the category of moderate problems.

Regarding self-care, no statistically significant differences were found between the sexes. Both groups had a higher frequency in the moderate problems category. However, with regard to usual activities, a statistically significant difference was found (*p* < 0.001), with women showing a significantly higher proportion of extreme problems (33.86%) compared to men (18.18%).

For pain/discomfort, statistically significant differences were observed between the sexes, both in the moderate problems subcategory (*p* = 0.04) and the extreme problems subcategory (*p* = 0.02). In the anxiety/depression dimension, statistically significant differences were also found (*p* = 0.02), with women showing a significantly higher proportion of no problems (36.22%) compared to men (18.18%). In the extreme problems subcategory, a statistically significant difference was observed between the sexes (*p* = 0.03).

While the EQ VAS demonstrated an average of 60.2, suggesting that the self-reported health status of the participants is predominantly considered moderately good, this average suggests a perceived quality of life that, although not exceptional, is at a satisfactory level for most participants.

## 4. Discussion

The objective of this study was to identify the main factors that influence the quality of life of caregivers in the context of home care for the elderly. The increase in longevity and the aging population have significantly impacted healthcare systems, generating a growing demand for prolonged home care. In this context, caregivers play a crucial role, providing assistance to individuals with chronic or disabling conditions [21]. However, this role can be challenging and stressful, with potential adverse consequences for the physical and emotional health of caregivers [22].

The results showed that most caregivers were women (92.03%) over 50 years of age and primarily the children of the care recipients (44.93%). Weekly visits from healthcare professionals were highly valued, emphasizing the importance of continuous and personalized home care.

These findings align with the international literature, which consistently highlights the feminization of caregiving and the predominance of first-degree family members in this role [23,24,25]. Female caregivers, particularly daughters, often assume this responsibility in contexts of chronic illness and disability, frequently without formal support.

Few studies have specifically evaluated the health of caregivers; our findings contribute to previous works, noting that the physical and mental health of caregivers is at risk [23,24,25]. Our analyses demonstrated statistically significant differences, which corroborate previous results. Barbosa et al. [25], using the EQ-5D-3L, observed that female home caregivers of the elderly are the majority and experience moderate or severe problems in all five dimensions. This can be attributed to the fact that they are often in a situation imposed by family members, accumulating multiple roles in the household and, most of the time, performing this role without financial support and family assistance [25,26,27,28].

Caregivers dedicated to long periods of care generally face compromised health outcomes, including higher mortality rates and disease prevalence [29]. Prolonged care imposes significant physical strain and tests caregivers’ psychological resilience, as evidenced by the participants’ reports. However, improvements in the physical environment can reduce stress and improve quality of life [30].

As demonstrated, many caregivers are older adults who face their own health problems and related symptoms [31], which can be exacerbated by caregiving [32]. While many studies address caregivers’ psychological symptoms, such as anxiety, depression, or sadness [33,34], few focus on caregivers’ physical symptoms, such as sleep disturbances, fatigue, and pain [35,36,37]. The impaired sleep reported by caregivers may be associated with the elderly’s nocturnal restlessness due to changes in the aging process and the onset of diseases [25,28].

In addition, challenging behaviors exhibited by older adults, such as aggression, agitation, and resistance to care, were also frequently reported by caregivers. These behavioral and psychological symptoms tend to intensify caregiver burden and should be addressed through integrated care strategies [38]. Studies highlight the effectiveness of non-pharmacological interventions and the role of multidisciplinary teams in managing aggression and agitation in the elderly. These findings reinforce the importance of incorporating such strategies into the routine of home care, promoting not only the well-being of the older adult but also the health of the caregiver [38,39].

Additionally, the continuous performance of caregiving tasks over extended periods generates significant stress, exacerbating physical conditions and triggering symptoms such as muscle tension and chronic pain [32], which may explain the pain reported by participants.

It is important to highlight that some caregivers in this study were older adults already facing health limitations, which may further complicate their caregiving role [28,32]. This dual vulnerability—being both a caregiver and an older adult—reinforces the need for tailored support strategies.

The findings of this study suggest a clear need for educational resources and emotional support for caregivers to alleviate the autonomous activities of their individual care and avoid misinterpreting their efforts to cope with and accommodate their condition [40,41,42]. Currently, family caregivers, although well intentioned, are not adequately supported in acquiring the necessary knowledge and skills to address the autonomous activities inherent in home care [43,44].

This study is relevant, as it addressed a diverse sample that offers a comprehensive representation of the caregiver population, considering variables such as age, gender, and relationship with the patient. This provides a more complete view of the characteristics, contributing to the external validation of the results. Additionally, the methodology allowed for an in-depth analysis of caregivers’ perceptions and the analysis of health-related quality of life. 

On the other hand, this study has limitations. This research was conducted in a single city, which may limit the applicability of the findings to other populations or geographical contexts. Future studies should consider longitudinal designs and interventions to support caregiver health, as well as comparative analyses across municipalities or countries. Integration with broader public health policies and caregiver training programs is essential to mitigate the burden of home caregiving and improve outcomes for both caregivers and care recipients.

Furthermore, despite the EQ-5D-3L being suitable for assessing general health-related quality of life in our sample, it may not fully capture the complexity of caregiver burden. Instruments like the Zarit Burden Interview (ZBI), validated in Brazil and widely used internationally, provide a more comprehensive assessment of emotional, physical, and financial strain experienced by informal caregivers [45]. Studies have shown that although the EQ-5D can reflect changes in health status, it often lacks sensitivity to specific psychological and social stressors addressed by the ZBI [46]. Therefore, future studies could benefit from using both tools to gain a more nuanced understanding of caregiver well-being and to better inform support strategies.

## 5. Conclusions

The majority of home caregivers are women over the age of 50, with a significant portion facing physical and emotional challenges due to the burden of caregiving. The constant presence of community health professionals, such as nurses and doctors, is highly valued by caregivers as it provides relief in the ongoing care at home. However, many caregivers report feelings of loneliness, sleep deprivation, and physical pain, especially in their arms, due to the constant effort of caring for debilitated patients. These conditions directly impact quality of life, highlighting the need for physical, psychological, and educational support.

Analysis of the EQ-5D dimensions indicated significant differences between genders, with women facing more difficulties in areas such as mobility, usual activities, pain, and anxiety/depression. The self-reported health status of the participants, measured by the EQ VAS, was moderately good (mean of 60.2), suggesting a reasonably positive perception but also pointing to the need for interventions to support these caregivers.

In this way, the data point to the urgent need for public policies that recognize and support the work of home caregivers, providing adequate physical, emotional, and social support, as well as continuous training and facilitated access to health services. Considering the aging population and the growing demand for home care, ensuring the well-being of these caregivers is essential for maintaining comprehensive, humanized, and sustainable care.

## Figures and Tables

**Table 1:** Sample distribution by categories of demographic and health variables.

	Variables	*n*	%
Gender			
	Masculine	11	7.97
	Feminine	127	92.03
Age range (years)			
	18–25 years	6	4.35
	26–30 years	15	10.87
	31–35 years	22	15.94
	40–50 years	27	19.57
	>50 years	68	49.28
Ethnicity/color			
	White	78	56.52
	Black	39	28.26
	Brown	21	15.22
Relationship with the patient			
	None	35	25.36
	Spouse	17	12.32
	Children	62	44.93
	Grandchildren	13	9.42
	Mother/Father	11	7.97
Working time as a caregiver			
	1–12 months	14	10.14
	13–24 months	38	27.54
	25–36 months	22	15.94
	37–48 months	16	11.59
	>50 months	48	34.78

**Table 2:** Themes and definitions emerging from the qualitative analysis of home care experiences.

Theme	Definition
Frequency and regularity of home visits	Reports on the consistent and weekly presence of healthcare professionals at the home, indicating a continuous pattern of care.
Importance of home care for treatment continuity	Caregivers’ perception of the positive impact of home visits on maintaining the patient’s health and addressing emergencies.
Emotional bond with healthcare professionals	Descriptions of the politeness, empathy, and emotional closeness of professionals with caregivers and patients, often compared to family members.
Feeling of loneliness and social isolation	Expressions of emotional distress due to a lack of social life, restricted freedom, and the burden of exclusive dedication to caregiving.
Physical and emotional overload	Reports of physical and psychological exhaustion related to the caregiving routine, sleep disruptions, muscular pain, and lack of adequate rest.

**Table 3:** Analysis of the EQ-5D dimension.

EQ-5D Dimension		Gender		
		Male *n* (%)	Female *n* (%)	*p*
Mobility	No problem	4 (36.36%)	38 (34.86%)	0.66
	Moderate problems	5 (45.45%)	49 (45.41%)	0.72
	Extreme problems	2 (18.18%)	40 (36.73%)	0.03 *
Self-care	No problem	3 (27.27%)	32 (25.20%)	0.52
	Moderate problems	5 (45.45%)	52 (40.95%)	0.08
	Extreme problems	3 (27.27%)	43 (33.86%)	0.05
Usual activities	No problem	5 (45.45%)	45 (35.43%)	0.05
	Moderate problems	4 (36.36%)	39 (30.71%)	0.06
	Extreme problems	2 (18.18%)	43 (33.86%)	<0.001 *
Pain/discomfort	No problem	3 (27.27%)	33 (25.98%)	0.05
	Moderate problems	6 (54.55%)	58 (45.67%)	0.04 *
	Extreme problems	2 (18.18%)	36 (28.35%)	0.02 *
Anxiety/depression	No problem	2 (18.18%)	46 (36.22%)	0.02 *
	Moderate problems	6 (54.55%)	60 (47.24%)	0.06
	Extreme problems	3 (27.27%)	21 (16.54%)	0.03 *

* The Chi-square test revealed a statistically significant difference (α = 0.05).

## Data Availability

The raw data supporting the conclusions of this article will be made available by the authors upon request.

## References

[1] Bloom D.E., Luca D.L. (2016). The global demography of aging: Facts, explanations, future. Handbook of the Economics of Population Aging.

[2] Wool E., Shotwell J.L., Slaboda J., Kozikowski A., Smith K.L., Abrashkin K., Rhodes K.V., Norman G.J., Pekmezaris R. (2019). A Qualitative Investigation of the Impact of Home-Based Primary Care on Family Caregivers. J. Frailty Aging.

[3] Mosquera I., Vergara I., Larrañaga I., Machón M., del Río M., Calderón C. (2015). Measuring the impact of informal elderly caregiving: A systematic review of tools. Qual. Life Res..

[4] Donnellan W.J., Bennett K.M., Soulsby L.K. (2014). What are the factors that facilitate or hinder resilience in older spousal dementia carers? A qualitative study. Aging Ment. Health.

[5] Stall N., Nowaczynski M., Sinha S.K. (2014). Systematic Review of Outcomes from Home-Based Primary Care Programs for Homebound Older Adults. J. Am. Geriatr. Soc..

[6] Shafir A., Garrigues S.K., Schenker Y., Leff B., Neil J., Ritchie C. (2016). Homebound Patient and Caregiver Perceptions of Quality of Care in Home-Based Primary Care: A Qualitative Study. J. Am. Geriatr. Soc..

[7] Bekdemir A., Ilhan N. (2019). Predictors of caregiver burden in caregivers of bedridden patients. J. Nurs. Res..

[8] Ohara Y., Iwasaki M., Motokawa K., Hirano H. (2021). Preliminary investigation of family caregiver burden and oral care provided to homebound older patients. Clin. Exp. Dent. Res..

[9] Schulz R., Beach S.R. (1999). Caregiving as a Risk Factor for Mortality. JAMA.

[10] Yang S.-Y., Fu S.H., Hsieh P.L., Lin Y.L., Chen M.C., Lin P.H. (2022). Improving the care stress, life quality, and family functions for family-caregiver in long-term care by home modification. Ind. Health.

[11] Omiya T., Kutsumi M., Fukui S. (2021). Work, Leisure Time Activities, and Mental Health among Family Caregivers of the Elder People in Japan. Healthcare.

[12] Mandani B., Hosseini S.A., Hosseini M.A., Noori A.K., Ardakani M.R.K. (2018). Perception of family caregivers about barriers of leisure in care of individuals with chronic psychiatric disorders: A qualitative study. Electron. Physician.

[13] Singh R., Kaur H. (2015). Barrier Free Environment and Universal Design: Approaches to Enhance the Functioning of People at Old Age.

[14] Rajão F.L., Martins M. (2020). Atenção Domiciliar no Brasil: Estudo exploratório sobre a consolidação e uso de serviços no Sistema Único de Saúde. Cien Saude Colet..

[15] Wachs L.S., Nunes B.P., Soares M.U., Facchini L.A., Thumé E. (2016). Prevalência da assistência domiciliar prestada à população idosa brasileira e fatores associados. Cad. Saude Publica.

[16] Lefèvre F., Lefèvre A.M.C., Teixeira J.J.V. (2000). O Discurso do Sujeito Coletivo: Uma Nova Abordagem Metodológica em Pesquisa Qualitativa.

[17] Lefèvre F., Lefèvre A.M.C. (2012). Pesquisa de Representação Social: Um Enfoque Qualiquantitativo.

[18] Genaro L.E., Marconato J.V., Tagliaferro E.P.D.S., Pinotti F.E., Valsecki Júnior A., Adas Saliba T., Rosell F.L. (2024). Home Care for the Elderly: An Integrated Approach to Perception, Quality of Life, and Cognition. Int. J. Environ. Res. Public Health.

[19] The EuroQol Group (1990). EuroQol—A new facility for the measurement of health-related quality of life. Health Policy.

[20] Devlin N.J., Brooks R. (2017). EQ-5D and the EuroQol Group: Past, Present and Future. Appl. Health Econ. Health Policy.

[21] Santos M., Cintra M.A.C.T., Monteiro A.L. (2015). Brazilian Valuation of EQ-5D-3L Health States: Results from a Saturation Study. Med. Decis. Making.

[22] Cassidy T., McLaughlin M. (2015). Psychological distress of female caregivers of significant others with cancer. Cogent Psychol..

[23] Haley W.E., LaMonde L.A., Han B., Narramore S., Schonwetter R. (2000). Family Caregiving in Hospice: Effects on Psychological and Health Functioning Among Spousal Caregivers of Hospice Patients with Lung Cancer or Dementia. Hosp. J..

[24] Williams A.L., McCorkle R. (2011). Cancer family caregivers during the palliative, hospice, and bereavement phases: A review of the descriptive psychosocial literature. Palliat. Support. Care.

[25] Barbosa L.C., Garbin C.A.S., Moimaz S.A.S., Saliba T.A. (2021). Cuidadores domiciliares de idosos: Qualidade de vida e práticas no processo de cuidar. Estud. Interdiscipl. Envelhec..

[26] Ascef B.D.O., Haddad J.P.A., Álvares J., Guerra Junior A.A., Costa E.A., Acurcio F.D.A., Silveira M.R. (2017). Qualidade de vida relacionada à saúde dos usuários da atenção primária no Brasil. Rev. Saude Publica.

[27] N’Goran A.A., Déruaz-Luyet A., Haller D.M., Zeller A., Rosemann T., Streit S., Herzig L. (2017). Comparing the self-perceived quality of life of multimorbid patients and the general population using the EQ-5D-3L. PLoS ONE.

[28] dos Reis E., Dourado V.Z., Guerra R.L.F. (2019). Qualidade de vida e fatores de riscos à saúde de cuidadoras formais de idosos. Estud. Interdiscip. Envelhec..

[29] Schulz R., Sherwood P.R. (2008). Physical and mental health effects of family caregiving. J. Soc. Work Educ..

[30] Carnemolla P., Bridge C. (2020). A scoping review of home modification interventions—Mapping the evidence base. Indoor Built Environ..

[31] Sambasivam R., Liu J., Vaingankar J.A., Ong H.L., Tan M.E., Fauziana R., Picco L., Chong S.A., Subramaniam M. (2019). The hidden patient: Chronic physical morbidity, psychological distress, and quality of life in caregivers of older adults. Psychogeriatrics.

[32] Shaffer K.M., Kim Y., Carver C.S., Cannady R.S. (2017). Effects of caregiving status and changes in depressive symptoms on development of physical morbidity among long-term cancer caregivers. Health Psychol..

[33] Adelman R.D., Tmanova L.L., Delgado D., Dion S., Lachs M.S. (2014). Caregiver burden: A clinical review. JAMA.

[34] Geng H.M., Chuang D.M., Yang F., Yang Y., Liu W.M., Liu L.H., Tian H.M. (2018). Prevalence and determinants of depression in caregivers of cancer patients: A systematic review and meta-analysis. Medicine.

[35] Fletcher B.A. (2008). Symptom experience of family caregivers of patients with cancer. Oncol. Nurs. Forum.

[36] Washington K.T., Oliver D.P., Smith J.B., McCrae C.S., Balchandani S.M., Demiris G. (2018). Sleep problems, anxiety, and global self-rated health among hospice family caregivers. Am. J. Hosp. Palliat. Med..

[37] Oechsle K., Ullrich A., Marx G., Benze G., Wowretzko F., Zhang Y., Dickel L.M., Heine J., Wendt K.N., Nauck F. (2020). Prevalence and predictors of distress, anxiety, depression, and quality of life in bereaved family caregivers of patients with advanced cancer. Am. J. Hosp. Palliat. Care.

[38] Grand J.H., Caspar S., Macdonald S.W. (2011). Clinical features and multidisciplinary approaches to dementia care. J. Multidiscip. Healthc..

[39] Vostrý M., Lanková B., Pešatová I., Fleischmann O., Jelínková J. (2022). Nonpharmacological Compensation of Aggressive Behavior of Individuals with Moderate Intellectual Disability and Behavioral Disorders—A Case Study. Int. J. Environ. Res. Public Health.

[40] de Araújo Reis L., de Souza Rocha T., Duarte S.F.P. (2014). Quedas: Risco e fatores associados em idosos institucionalizados. Rev. Baiana Enferm..

[41] Cott C.A., Tierney M.C. (2013). Acceptable and unacceptable risk: Balancing everyday risk by family members of older cognitively impaired adults who live alone. Health Risk Soc..

[42] Car L.T., El-Khatib M., Perneczky R., Papachristou N., Atun R., Rudan I., Car J., Vincent C., Majeed A. (2017). Prioritizing problems in and solutions to homecare safety of people with dementia: Supporting carers, streamlining care. BMC Geriatr..

[43] Häikiö K., Sagbakken M., Rugkåsa J. (2019). Dementia and patient safety in the community: A qualitative study of family carers’ protective practices and implications for services. BMC Health Serv. Res..

[44] Hung L., Hudson A., Gregorio M., Jackson L., Mann J., Horne N., Berndt A., Wallsworth C., Wong L., Phinney A. (2021). Creating Dementia-Friendly Communities for Social Inclusion: A Scoping Review. Gerontol. Geriatr. Med..

[45] Seng B.K., Luo N., Ng W.Y., Lim J., Chionh H.L., Goh J., Yap P. (2010). Validity and reliability of the Zarit Burden Interview in assessing caregiving burden. Ann. Acad. Med. Singap..

[46] Carod-Artal F.J., Mesquita H.M., Ziomkowski S., Martinez-Martin P. (2013). Burden and health-related quality of life among caregivers of Brazilian Parkinson’s disease patients. Park. Relat. Disord..

